# Bilateral Mandibular Condylar Fractures

**DOI:** 10.31662/jmaj.2024-0378

**Published:** 2025-03-07

**Authors:** Koji Tajima

**Affiliations:** 1 Division of Surgery, Kokuho Nokami Kosei Sogo Hospital, Wakayama, Japan

**Keywords:** mandibular condylar fracture, trauma, primary care

A 95-year-old woman presented with facial injuries after a fall from her car. Her denture was broken. Lacerations on her chin and lower lip were identified and sutured.

Subsequently, the patient reported pain around the right temporomandibular joint. Although there was no wound, mild tenderness was noted at the joint, with no restriction in mouth opening. Computed tomography revealed bilateral mandibular condylar fractures (MCFs) (Figure 1).

**Figure 1.**
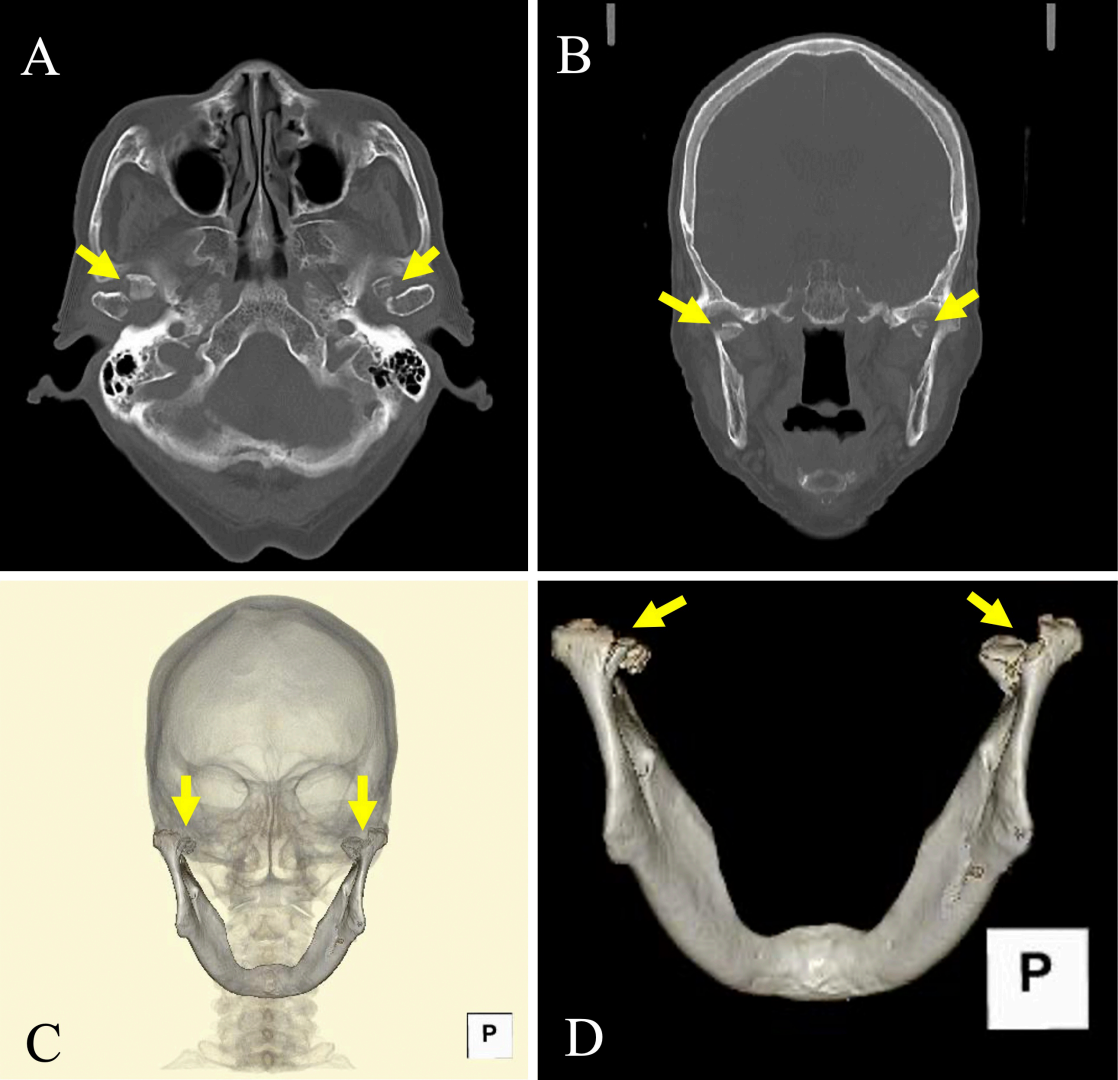
Computed tomography images. (A) Axial image. (B) Coronal image. (C) Three-dimensional (3D)-reconstructed image. (D) Mandibular 3D-reconstructed image. Arrows indicate fracture lines.

Kannari et al. ^(1)^ reported that 20% of mandibular fractures were undiagnosed at the first healthcare visit, with an average delay of 11.3 days to diagnosis. Pain is the most common symptom of MCF, reported in 78.5% of cases ^(1)^.

Limited mouth opening was observed in 43.0% of patients with mandibular fractures ^(1)^. Pain during mouth opening can also contribute to restricted movement ^(2)^.

In patients who are edentulous, the interridge distance between the upper and lower gums should be measured. However, this measurement may be overestimated owing to the absence of incisors compared with patients who are dentulous. The cut-off value for the limitation of mouth opening is 35 mm in patients who are dentulous and 40 mm in those who are edentulous ^(3)^.

In this case, the broken denture suggested that significant pressure was exerted on the mandible. Even when symptoms are subtle, imaging studies should be performed if strong force has been applied. Evaluating facial X-rays for fractures can be challenging for primary care clinicians; therefore, computed tomography should be considered during the initial consultation.

Clinicians should, therefore, attend to painless and bilateral MCFs in patients with facial injuries, particularly in older individuals.

## Article Information

### Conflicts of Interest

None

### Acknowledgement

The author thanks the patient for their kind cooperation.

### Author Contributions

KT: Conception and design of the study, acquisition of data, drafting the article, and final approval of the version to be submitted.

### Approval by Institutional Review Board (IRB)

IRB approval was not required in this study.

### Informed Consent

Written consent has been obtained from the patient to publish the information, including the photographs.
